# The impact of time from injury to surgery on the risk of neuropathic pain after traumatic spinal cord injury

**DOI:** 10.1186/s13018-023-04355-7

**Published:** 2023-11-11

**Authors:** Tengbin Shi, Zhengxi Yu, Zhi Chen, Dingwei Wu, Zhenyu Wang, Wenge Liu

**Affiliations:** 1 Fujian Medical University Union Hospital, No. 29 Xinquan Road, Fuzhou, 350000 China; 2 Department of Orthopedics, Fujian Medical University Union Hospital, No. 29 Xinquan Road, Fuzhou, 350000 China; 3 Department of Minimally Invasive Spinal Surgery, The Affiliated Hospital of Putian University, No. 999 Dongzhen East Road, Licheng District, Putian, 351100 China

**Keywords:** Traumatic spinal cord injury, Neuralgia, Pain, Neuropathic pain, Surgery timing

## Abstract

Traumatic spinal cord injury (SCI) is a devastating neurological disorder often accompanied by neuropathic pain (NeP), significantly affecting patients' quality of life. This retrospective study aimed to investigate the impact of the time from injury to surgery on the development of NeP following traumatic SCI. Medical records of patients with traumatic SCI who underwent surgical intervention between January 2017 and January 2021 at two specialized centers were reviewed. Variables associated with NeP including demographics, injury profiles, medical history, surgical details, and pain assessments were investigated. Independent risk factors related to NeP were identified using multivariate logistic regression analysis. A total of 320 patients met the inclusion criteria, with 245 (76.6%) being male and a mean age of 56.5 ± 13.2 years. NeP was identified in 48.4% of patients (155 of 320). The multivariate analysis identifies age at injury, Injury Severity Score, and the neurological level of injury as independent risk factors for the development of NeP in both AIS A and AIS B, C, and D subgroups. Additionally, a significant association between the time from injury to surgery and NeP was observed in AIS B, C, and D patients, while no such association was found in AIS A patients. This study highlights the benefits of early and ultra-early surgical intervention in preventing NeP in patients with incomplete traumatic SCI (AIS B, C, and D), underscoring the importance of optimizing surgical timing to improve patient outcomes. Prospective studies are warranted to establish evidence-based surgical guidelines for managing traumatic SCI and preventing NeP effectively.

**Supplementary Information:**

The online version contains supplementary material available at 10.1186/s13018-023-04355-7.

## Introduction

Traumatic spinal cord injury (SCI) poses a significant clinical and public health challenge, resulting in substantial morbidity and disability [1, 2]. Despite advances in understanding the pathophysiology and secondary mechanisms of SCI, few effective treatments are currently available [3]. Alongside motor and sensory deficits, individuals with SCI frequently endure the burden of neuropathic pain (NeP) [4–6], which is characterized by spontaneous pain, hyperalgesia, abnormal pain, and paranesthesia [7]. The overall point prevalence rates for NeP in these patients were established at 53% [8], and recent data state that 30–50% of SCI patients suffer from NeP within a year after injury [9, 10], a significantly higher percentage compared to the general population (7–10%) [11, 12]. NeP following SCI can manifest as at-level pain (corresponding to the segment of injury) and below-level pain (corresponding to segments below the injury site) where at-level pain may consist of both peripheral and central neuropathic pain, while below-level pain is a central neuropathic pain condition. These conditions can manifest as spontaneous or evoked pain, featuring sensory deficits, allodynia, or hyperalgesia within the pain distribution, and described as hot/burning, tingling, pricking, pins and needles, sharp, shooting, squeezing, painful cold, and electric shock-like sensations [4, 5, 7, 13].

Severe neuropathic pain after SCI is recognized as one of the most distressing, debilitating, and incapacitating conditions, often leading to a lifelong and significant negative impact on a person's ability to participate in activities, recreation, and entertainment. This condition results in a compromised quality of life (QOL), long-term reliance on drug abuse, sleep disturbances, anxiety, depression, and reduced functionality [5, 6, 8, 12, 14]. Managing SCI NeP is a significant and unmet clinical challenge due to its refractory and chronic nature, frequently resisting conventional treatments. Regrettably, there is a glaring lack of research into the underlying mechanisms of SCI NeP. Therefore, a deeper understanding of these mechanisms and identifying high-risk factors are essential to enhance patient care and develop effective and rational treatment strategies.

The proper time of surgical intervention of traumatic SCI has been an area of ongoing research. Surgeons have increasingly recognized the significance of secondary injury in SCI, leading to the growing recognition of the concept of “time is spine.” This concept supports the notion that early or urgent surgical intervention, including decompression and stabilization, can be safe and effective for traumatic SCI, restoring blood flow improving perfusion, and potentially mitigating the course of secondary injury [3, 15–17]. Recent evidence also suggests that early surgical treatment may have positive effects on functional outcomes [16, 18, 19]. Dvorak et al. [18] assessed the effect of an early surgical procedure (< 24 h from injury) on motor recovery and length of stay (LOS) of 470 individuals with traumatic SCI. Their findings demonstrated that surgery performed within 24 h of the injury improved motor neurological recovery and reduced LOS in cases of incomplete acute traumatic SCI in the cervical, thoracic, or thoracolumbar spine.

Despite this progress in understanding the benefits of early surgical intervention for traumatic SCI, there remains a lack of data regarding its influence on the development of neuropathic pain after traumatic SCI. Hence, the current study aimed to address two objectives: (1) investigate the prevalence and status of NeP in patients with traumatic SCI, utilizing the Douleur Neuropathique 4 Questions (DN4) questionnaire from baseline to the final neurological follow-up and (2) determine the extent to which the time from injury to surgery affects the development of NeP after acute traumatic SCI. This research seeks to shed light on an important aspect of SCI management and contribute to better understanding and optimizing treatment strategies for individuals suffering from this condition.

## Methods

### Study design

Consecutive adult patients (aged > 18 years) admitted to two specialized tertiary rehabilitation hospitals specifically catering to acute traumatic SCI (C2-L2, AIS A-D) were retrospectively included between 2017.01 and 2021.01. Only patients who received surgical intervention within one week after sustaining the injury possessed complete medical records, had preoperative and postoperative pain assessments available, and underwent follow-up for a minimum of 12 months were considered eligible for inclusion. Exclusions were made for patients with non-traumatic spinal cord injuries (excluding single-event ischemia) and those who deceased during the primary rehabilitation period in the hospital. Furthermore, patients with peripheral nerve injury, diabetes mellitus, tumors, severe spasticity (Ashworth grade 3 or higher), or any other co-morbidity that could impact the neuraxial or peripheral nervous system were not included in the study. The study's protocols were duly approved by the relevant institutional research ethics board, and informed consent was waived.

A comprehensive set of demographic data, encompassing age, gender, age at injury, education level, smoking, and alcohol habits, as well as the patient's past medical history and the Charlson Co-morbidity Index (CCI), were collected. Relevant SCI-related characteristics such as the injury level, time and mechanism of injury, time from injury to surgery, and Injury Severity Score (ISS) were meticulously collected. Regarding pain-related data, information on the severity and nature of pain, its specific location, factors that alleviate or exacerbate it, its impact on daily activities, frequency of accompanying symptoms, and treatments employed were diligently documented. Length of hospital stays (LOS) and hospitalization expenses were also recorded. For the analysis of time from injury to surgery, clinically relevant time intervals were chosen, with 8 and 24 h being the primary cut-offs. These specific timeframes have been frequently studied in patients with traumatic SCI [20].

### Neurological examination and pain assessments

A neurological examination was performed following the International Standards for Neurological Classification of Spinal Cord Injury (ISNCSCI) [21] at a detailed initial or baseline. The severity of the injury was determined based on the American Spinal Injury Association Impairment Scale (AIS) grade, which assigns a grade of A, B, C, or D to the injury level. The lesion level was identified as the most rostral spinal segment with intact sensorimotor function. To assess the locations of pain, participants used a body diagram to mark the areas where they experienced the most pain. The pain was classified according to the International Spinal Cord Injury Pain (ISCIP) classification [4], a mechanism-based classification that distinguishes between nociceptive pain, neuropathic pain, other pain, and unknown pain in patients with SCI, which has been validated by the American Spinal Injury Association (ASIA) and the International Spinal Cord Society (ISCoS).

For the purposes of this study, SCI neuropathic pain (NeP) was defined as pain with a positive DN4 (4/10) rating, occurring at or below the level of injury and involving areas with sensory abnormalities as assessed by the DN4 questionnaire and the ISNCSCI examination. In this study, the Chinese version of the DN4 questionnaire [22] was independently administered by two trained doctors to characterize the individual’s worst pain at baseline and 3 days, 1 month, 3 months, 6 months, and 12 months post-SCI. The DN4 was initially developed as a screening tool for neuropathic pain, which is a 10-item questionnaire based on sensory descriptors and an exam, the specificity for detecting neuropathic pain is evaluated at 82.9% and sensitivity at 89.9% [23]. The first seven items access specific pain descriptors (burning, painful cold, electric shocks) as well as paresthesia and dysesthesias (tingling, pins and needles, numbness, itching). The remaining three items are derived from a targeted bedside sensory examination in the pain area (touch hypoesthesia, pinprick hypoalgesia, dynamic mechanical allodynia). For scoring, each positive item is assigned one point, and each negative item is assigned zero points, with a total score range of 0 to 10, and a score of ≥ 4 indicates that the pain may be neuropathic. The questionnaire has previously been used in individuals with chronic and acute neuropathic pain [24, 25]. Pain intensity was evaluated using the Numerical Rating Scale (NRS) and the Short-form McGill Pain Questionnaire (SF-MPQ).

All medical records and questionnaires were systematically recorded and reviewed by two independent authors/observers, and any discrepancies were resolved through consensus or consultation with senior co-authors.

### Data analysis

Data were presented as mean ± standard deviation (SD) (or median with interquartile range (IQR), where applicable) for continuous variables and frequencies for categorical variables. The normality of distribution was tested by the Shapiro−Wilk test. In the complete cohort, using the Mann–Whitney U test for nonparametric variables, Student’s t test for parametric variables, and the χ2 test for categorical variables.

Based on the significantly different neural recovery patterns and neurological outcomes between patients with complete injury (AIS A) and patients with incomplete injury (AIS B, C, and D) [26, 27], we chose to analyze AIS A and AIS B, C, and D subgroups separately. The risk factors for neuropathic pain were investigated by univariate regression analysis, and the results were controlled using a binary logistic regression model. Binary logistic regression analysis (forward stepwise LR method) was employed to control the results. Variables that exhibited statistical significance (*p* value < 0.1) in the univariate regression analysis or were deemed clinically relevant were included in the multivariate analysis. The Box-Tidwell test was applied to evaluate the assumption of linearity in the logit for continuous variables. Multicollinearity was assessed using the variance inflation factor in a multiple regression model with the same variables. The results were expressed as odds ratios (ORs) with their corresponding 95% CIs (confidence intervals). The sample size in our study met the demands of events per variable during logistic regression analysis. A two-tailed *p* value < 0.05 was considered statistically significant. The statistical analyses were conducted using SPSS 26.0 (IBM, Armonk, NY, USA), while GraphPad Prism (Version 8) was utilized for data visualization.

## Results

**Fig. 1:**
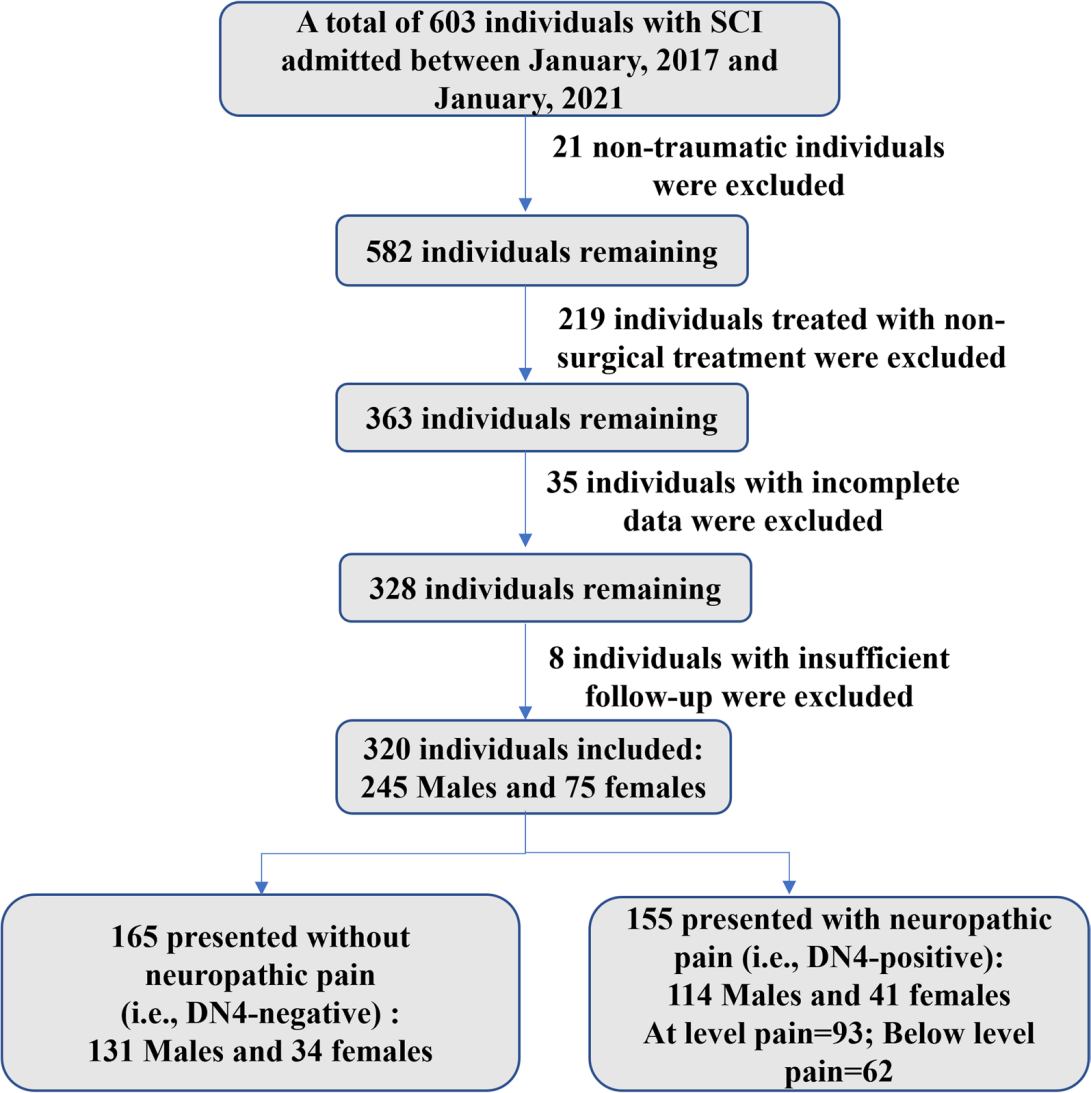
Participant flowchart

From a total population of 363 patients who met our inclusion criteria, 35 were excluded due to incomplete data, and 8 were excluded due to insufficient follow-up, leaving 320 individuals with complete datasets in the final analysis (Fig. 1). The mean age of the overall study population was 56.5 ± 13.2 years and ranged between 18.0 and 89.0 years. The demographic characteristics and injury profiles are summarized in Table 1. Participants of this study had been injured for 5.6 ± 3.8 years and were 57.1 ± 13.5 years old when injured. Most participants were male (245/320, 76.6%), and falls were the most common injury mechanism (163/320, 50.9%) and then Traffic Accident (114/320, 35.6%), and cervical injuries were the most common (237/320, 74.1%). Participants presented with sensory and motor complete (AIS A, *n* = 112), sensory incomplete (AIS B, *n* = 45), and motor incomplete (AIS C/D, *n* = 163) lesions. Neurological levels of injury were: high cervical (*n* = 101), low cervical (*n* = 136), thoracic (*n* = 36), and thoracolumbar (*n* = 47). The mean time from injury to surgery was 44.2 ± 29.7 h, 9.7% (31/320) of patients undergoing surgical intervention within 8 h from the time of injury, 20% (64/320) of patients undergoing surgical intervention within 24 h from the time of injury and the remaining 70.3% (225/320) having a surgical procedure after 24 h and up to 150 h after injury. Additional file 1: Table S1 shows the breakdown of participants based on their injury severity and anatomical level of injury.

**Table 1:** Comparison of baseline and injury characteristics between patients with or without neuropathic pain after traumatic spinal cord injury

Characteristics	All (*N* = 320)	Neuropathic pain (DN4-positive, *N* = 155)	No neuropathic pain (DN4-negative, *N* = 165)	P value
Age (y)	56.5 (13.2)	56.8 (11.8)	56.3 (14.6)	0.714*
Sex				0.217^†^
Female	75 (23.4)	41 (26.5)	34 (20.6)	
Male	245 (76.6)	114 (73.5)	131 (79.4)	
Age at injury (y)	57.1 (13.5)	58.8 (14.8)	55.6 (11.9)	0.031*
Time post-injury (y)	5.6 (3.8)	5.2 (3.5)	5.7 (3.6)	0.364*
Body mass index (kg/m^2^)				0.660^†^
< 24	132 (41.3)	62 (40.0)	70 (42.4)	
≥ 24	188 (58.8)	93 (60.0)	95 (57.6)	
Current smoker	72 (22.5)	35 (22.6)	37 (22.4)	0.973^†^
Etiology				0.513^†^
Falls	163 (50.9)	74 (47.7)	89 (53.9)	
Traffic accident	114 (35.6)	62 (19.4)	52 (31.5)	
Violence	16 (5.0)	7 (4.5)	9 (5.5)	
Others	27 (8.4)	12 (7.8)	15 (9.1)	
ISS				**0.034**^†^
< 16	238 (74.4)	107 (69.0)	131 (79.4)	
≥ 16	82 (25.6)	48 (31.0)	34 (20.6)	
AIS grade				**0.026**^†^
A	112 (35.0)	67 (43.2)	45 (27.3)	
B	45 (14.1)	18 (11.6)	27 (16.4)	
C	78 (24.4)	32 (20.6)	46 (27.9)	
D	85 (26.6)	38 (24.5)	47 (28.5)	
Neurological level of injury				0.691^†^
High cervical (C1–C4)	101 (31.6)	50 (32.3)	51 (30.9)	
Low cervical (C5–T1)	136 (42.5)	68 (43.9)	68 (41.2)	
Thoracic (T2–T10)	36 (11.2)	14 (9.0)	22 (13.3)	
Thoracolumbar (T11–L2)	47 (14.9)	23 (14.8)	24 (14.5)	
Baseline neurological examination time (hours post-injury):	13.8 (10.2)	12.6 (10.1)	14.3 (11.8)	0.595*
Time from injury to surgery, hours	44.2 (29.7)	45.5 (29.6)	38.4 (25.6)	0.023*
Treated with high-dose methylprednisolone	126 (39.4)	63 (40.6)	48 (29.1)	**0.030**^†^
LOS, days	26.0 (20.1)	26.6 (22.8)	25.4 (17.4)	0.594*
Total cost, ¥	95,378.2 (62,753.9)	92,904.9 (61,788.1)	97,996.2 (63,857.2)	0.471*
Follow-up duration, months	36.5 (26.8)	35.9 (28.2)	36.6 (26.1)	0.689*

The bold font indicates that the corresponding *P* value is less than 0.05

*ISS* Injury Severity Score, *AIS* American Spinal Injury Association Impairment Scale, *LOS* length of stay

^*^Independent-sample *t* test, ^†^Pearson chi-squared test or Fisher's exact test

**Fig. 2:**
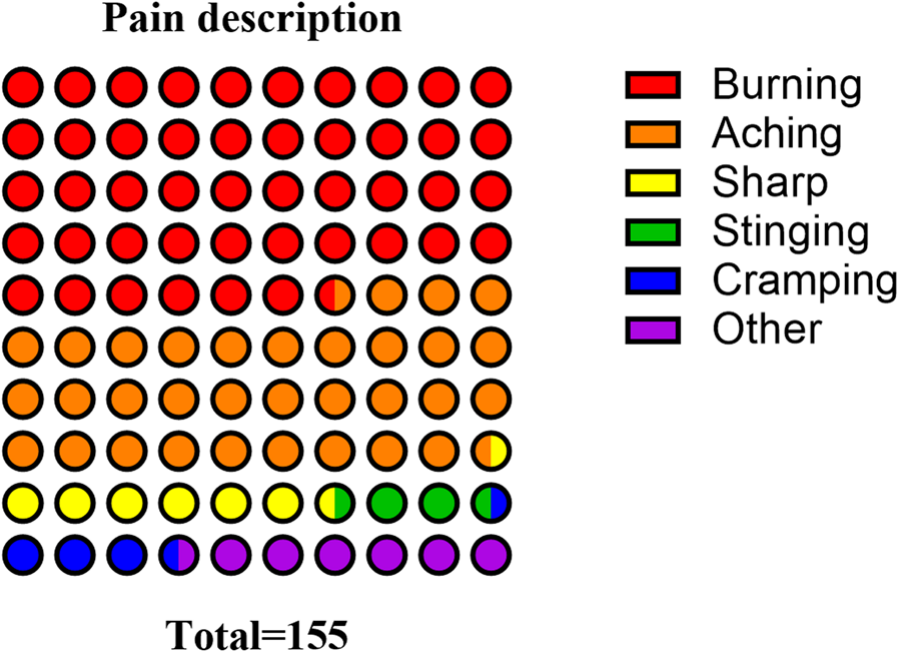
Neuropathic pain description

**Fig. 3:**
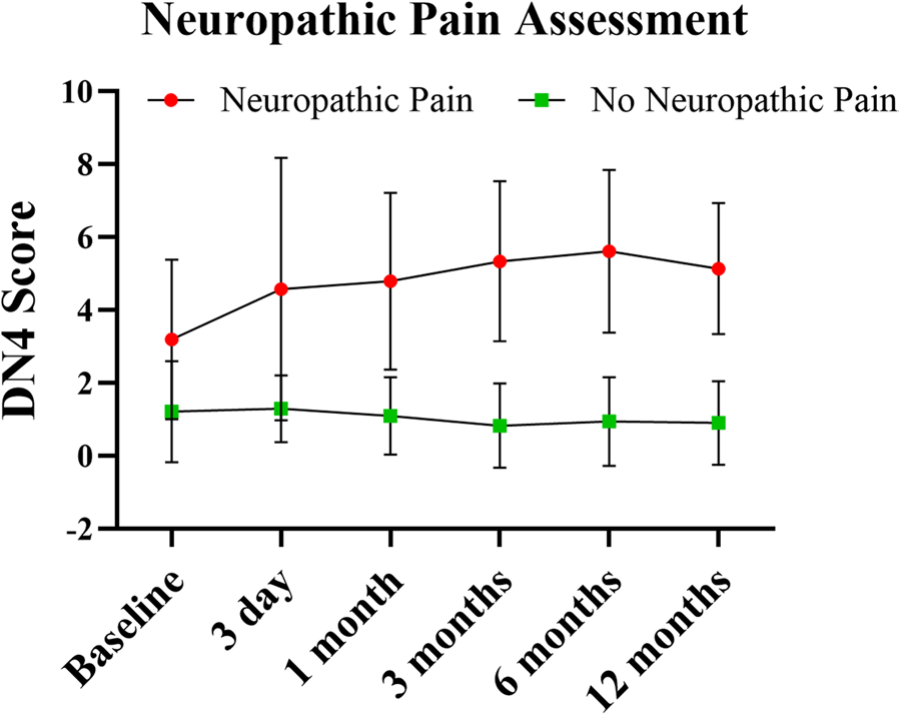
Assessment of neuropathic pain using Douleur Neuropathique 4 Questions (DN4) questionnaire at baseline and 3 days, 1 month, 3 months, 6 months, 12 months post-SCI

Out of 320 individuals, 155 (48.4%) presented with neuropathic pain (i.e., DN4-positive) as their most severe pain, while at-level NeP could be identified in 60.0% (93/155) of these individuals. The timeline of NeP occurrence was also investigated, 32.9% (51/155) of the individuals developed NeP (DN4-positive) within 3 months post-injury and the remaining 67.1% (104/155) presented with possible neuropathic pain at the chronic stage (> 3 months). Mechanical allodynia was present in 22.6% of individuals, and thermal allodynia was present in 27.1% of individuals (Table 2). Figure 2 shows the pain description of NeP, which mostly has a burning and aching character. Figure 3 shows the assessment of NeP at different times after SCI surgery, showing a significantly higher DN4 score at 12 months post-SCI (5.1 ± 1.8 vs. 0.9 ± 1.1, *P* < 0.001; Table 2).

**Table 2:** Neuropathic pain characteristics of traumatic SCI

	Neuropathic pain (DN4-positive, *N* = 155)	No neuropathic pain (DN4-negative, *N* = 165)	P value
Neuropathic pain			
At-level	93 (60.0)		
Below-level	62 (40.0)		
Above-level	0		
Trauma-pain onset interval			
≤ 3 months	51 (32.9)		
> 3 months	104 (67.1)		
DN4 score	5.1 (1.8)	0.9 (1.1)	< .001*
Mechanical allodynia	35 (22.6)		
Thermal allodynia	42 (27.1)		
Pain intensity, NRS	6.2 (3.8)	1.2 (1.3)	< .001*
Spasticity	112 (72.3)	46 (27.9)	** < .001**^†^
SF-MPQ	16.3 (9.8)	0.8 (0.9)	< .001*
Pain medication			
Antidepressants pregabalin/gabapentin)	92 (59.4)		
Antiepileptics	67 (43.2)		
NSAIDs	106 (68.4)		
Opioids	45 (29.0)		
Other	35 (22.6)		
None	26 (16.8)		

The bold font indicates that the corresponding *P* value is less than 0.05

*DN4* Douleur Neuropathique 4 Questions, *NRS* Numerical Rating Scale, *SF-MPQ* Short-Form McGill Pain Questionnaire Scores, *NSAIDs* nonsteroidal anti-inflammatory drugs

*Independent-sample *t* test, ^†^Pearson chi-squared test or Fisher's exact test

Patients with NeP were significantly older than those without NeP (58.8 years vs. 55.6 years, *p* = 0.031; Table 1) at the time of injury. Patients with NeP also showed a higher (worse) ISS score (*p* = 0.034) and more severe neurological injury (as measured by admission AIS grade, *p* = 0.026) than those without NeP. Patients with NeP recorded a significantly longer time from injury to surgery compared to patients without NeP (*P* = 0.023). It is worth noting that patients with NeP seem more likely to receive high-dose methylprednisolone therapy at the acute phase (*p* = 0.030). Differences were not observed in sex, BMI, etiology, and Neurological level of injury (*p* > 0.05).

After the univariate logistic regression analysis, NeP in AIS A patient was significantly associated with age at injury (*p* = 0.037), ISS score (*p* = 0.020), neurological level of injury (*p* = 0.025) and treated with high-dose methylprednisolone (*p* = 0.008, Table 3). NeP in AIS B, C, and D patients was significantly associated with age at injury (*p* = 0.012), ISS score (*p* = 0.008), neurological level of injury (*p* = 0.038), treated with high-dose methylprednisolone (*p* = 0.007), and time from injury to surgery (*p* = 0.024, Table 3).

**Table 3:** Risk factors for neuropathic pain in complete or incomplete SCI according to American Spinal Injury Association Impairment Scale: univariate logistic regression analysis

	AIS A grade			AIS B, C and D grade		
	Odds ratios	95% confidence interval	p value	Odds ratios	95% confidence interval	p value
Age (y)			0.576			0.703
≤ 40	–	–		–	–	
41–60	0.684	(0.183, 2.548)	0.571	0.588	(0.170, 2.039)	0.403
> 60	1.389	(0.282, 6.835)	0.686	0.603	(0.170, 2.138)	0.433
Sex						
Male	1.344	(0.541, 3.340)	0.525	0.744	(0.410, 1.461)	0.430
Female	–	–		–	–	
Age at injury (y)			**0.037**			**0.012**
≤ 40	–	–		–	–	
41–60	0.556	(0.164, 1.883)	0.345	6.429	(1.420, 29.10)	0.016
> 60	1.778	(0.483, 6.547)	0.387	9.462	(2.059, 43.48)	0.004
Time post-injury (y)	0.859	(0.825, 2.282)	0.625	1.153	(0.965, 1.282)	0.825
Body Mass Index(kg/m^2^)						
< 24	0.936	(0.953, 1.071)	0.863	0.800	(0.459, 1.395)	0.432
≥ 24	–	–	–	–	–	–
Current smoker	0.859	(0.466, 1.582)	0.744	1.004	(0.515, 1.959)	0.990
Highest level of education			0.825			0.098
Illiterate	1.158	(0.282, 4.749)	0.839	0.262	(0.065, 1.055)	0.060
Pre–high school	0.681	(0.254, 1.823)	0.445	0.960	(0.440, 2.095)	0.918
High school	0.891	(0.321, 2.468)	0.824	0.553	(0.274, 1.115)	0.098
College and above	–	–	–	–	–	–
Etiology			0.326			0.579
Falls	0.635	(0.145, 2.783)	0.547	1.302	(0.453, 3.742)	0.625
Traffic accident	1.136	(0.240, 5.390)	0.872	1.805	(0.615, 5.294)	0.282
Violence	0.300	(0.041, 2.200)	0.236	2.000	(0.366, 10.919)	0.423
Others	–	–		–	–	
ISS			**0.020**			**0.008**
< 16	–	–		–	–	
≥ 16	2.525	(1.157, 5.507)		3.171	(1.350, 7.452)	
Neurological level of injury			**0.025**			**0.038**
High cervical (C1–C4)	3.542	(1.350, 9.291)	0.010	2.340	(1.037, 5.278)	0.041
Low cervical (C5–T1)	1.366	(0.514, 3.628)	0.531	2.509	(1.215, 5.184)	0.013
Thoracic/thoracolumbar (T2–L2)	–	–		–	–	
Baseline neurological examination time (hours post-injury):	1.636	(0.823, 2.326)	0.845	1.159	(0.859, 2.153)	0.658
Time from injury to surgery			0.696			**0.024**
≤ 8 h	0.703	(0.185, 2.676)	0.605	0.221	(0.062, 0.795)	0.021
8–24 h	1.255	(0.550, 2.862)	0.589	0.503	(0.224, 1.131)	0.096
> 24 h	–	–		–	–	
Treated with high-dose methylprednisolone	2.870	(1.314, 6.266)	**0.008**	2.275	(1.249, 4.143)	**0.007**

The bold font indicates that the corresponding *P* value is less than 0.05

*ISS* Injury Severity Score, *AIS* American Spinal Injury Association Impairment Scale

Stepwise multivariate logistic regression analysis identified that NeP in AIS A patients was associated with a higher age at injury (*p* = 0.040, Table 4), higher ISS score (*p* = 0.023), and neurological level of injury (*p* = 0.013). NeP in AIS B, C, and D patients was consistently associated with a higher age at injury (*p* = 0.025), higher ISS score (*p* = 0.006), longer time from injury to surgery (*p* = 0.014), and history of high-dose methylprednisolone therapy (*p* = 0.002). Time from injury to surgery was not significantly associated with NeP in AIS A patients, however, AIS B, C, and D patients who had a surgical procedure performed within 8 h (ultra-early) of injury had significantly less NeP than those with surgery performed after 24 h (late) (*p* = 0.021), and patients who had a surgical procedure performed within 24 h (early) of injury had significantly less NeP than those with surgery performed after 24 h (late) (*p* = 0.039).

**Table 4:** Risk factors for neuropathic pain in complete or incomplete SCI according to American Spinal Injury Association Impairment Scale: multivariate logistic regression analysis

	AIS A grade			AIS B, C and D grade		
	Odds ratios	95% Confidence interval	*p* value	Odds ratios	95% Confidence interval	*p* value
Age at injury (y)			**0.040**			**0.025**
≤ 40	–	–		–	–	
41–60	1.045	(0.170, 0.817)	0.016	5.997	(1.186, 30.329)	0.030
> 60	1.372	(0.170, 0.817)	0.020	9.093	(1.754, 47.127)	0.009
ISS						
< 16	–	–		–	–	
≥ 16	2.960	(1.162, 7.540)	**0.023**	3.837	(1.482, 9.937)	**0.006**
Neurological level of injury			**0.013**			0.077
High cervical (C1–C4)	6.595	(1.809, 24.045)	0.004	2.428	(0.963, 6.122)	0.060
Low cervical (C5–T1)	1.587	(0.516, 4.881)	0.421	2.367	(1.070, 5.240)	0.034
Thoracic/Thoracolumbar (T2–L2)	–	–		–	–	
Time from injury to surgery			0.278			**0.014**
≤ 8 h	0.280	(0.056, 1.396)	0.121	0.148	(0.029, 0.751)	0.021
8–24 h	0.906	(0.291, 2.826)	0.865	0.375	(0.148, 0.950)	0.039
> 24 h	–	–		–	–	
Treated with high-dose methylprednisolone	2.541	(0.991, 6.517)	0.052	3.071	(1.525, 6.185)	**0.002**

Model: adjusted for age at injury, ISS score, neurological level of injury, time from injury to surgery, and treated with high-dose methylprednisolone. The bold font indicates that the corresponding *P* value is less than 0.05

*ISS* Injury Severity Score, *AIS* American Spinal Injury Association Impairment Scale

## Discussion

The present retrospective study, conducted across two centers, sought to probe into the occurrence of NeP in patients afflicted with traumatic SCI and its potential association with the time from injury to surgery. Several factors, including age at injury, Injury Severity Score, and the neurological level of injury, exhibited links to the development of NeP in both complete and incomplete injury subgroups. Interestingly, in the incomplete SCI subgroups (AIS B, C, and D), delayed surgical intervention was identified as a key contributor to an augmented risk of NeP. These findings furnish compelling evidence reinforcing the advantages of early surgical intervention, notably ultra-early surgery, in mitigating the incidence of NeP after traumatic SCI.

The reported incidence rate of NeP following traumatic SCI displays a wide range of 25–84% [8, 10, 28, 29]. Such variation is primarily attributed to diverse research methodologies, substantial heterogeneity in population characteristics, and the adoption of different definitions and diagnostic criteria for NeP. The present study identified that 48.4% (155 of 320) of patients with traumatic SCI suffered from NeP using the DN4 Neuropathological Pain Scale, which has been reported as a clinical and validated diagnostic tool used for screening acute and chronic neuropathic pain [24, 25, 30].

Moreover, within the subset of patients experiencing NeP, a significant 60.0% were found to exhibit at-level NeP. Notably, and regarding the timeline of NeP occurrence, a substantial proportion (32.9%) of individuals developed NeP within 3 months post-injury, while the majority (67.1%) presented with possible neuropathic pain at the chronic stage (> 3 months). These findings underscore the critical importance of comprehending the temporal evolution of NeP in SCI patients, a factor that can significantly inform clinical management and intervention strategies. Furthermore, our analysis identified several factors consistently associated with the development of NeP in both AIS A and AIS B, C, and D subgroups. Specifically, older age at injury, higher Injury Severity Score (ISS), and more severe neurological injury were all found to be significantly linked to an elevated likelihood of NeP. These associations shed light on the substantial influence of injury severity and patient characteristics on the development of NeP.

The principal focus of this study was to explore the potential impact of the time from injury to surgery on the occurrence of NeP in patients with SCI. Intriguingly, we observed that patients afflicted with NeP exhibited a significantly prolonged duration between injury and surgical intervention compared to those without NeP. This observation suggests a plausible association between delayed surgical intervention and the development of NeP. Earlier research has also reported a correlation between surgical timing and improved clinical outcomes in SCI patients [22, 31, 32]. For instance, Middendorp et al. [22] conducted a systematic review and meta-analysis encompassing studies evaluating the impact of the timing of spinal surgery after traumatic SCI. Their findings highlighted that early spinal surgery was significantly linked to higher total motor and neurological improvement in comparison with late surgery. The influence of early surgery or the timing of surgery on postoperative neuropathic pain in patients with traumatic SCI remains unexplored, representing a critical knowledge gap in the field. In this current investigation, a multivariate logistic regression analysis has revealed a significant association between the time from injury to surgery and NeP in AIS B, C, and D patients, while we failed to demonstrate that the time from injury to surgery affects the occurrence of NeP after injury in AIS A (complete injury) patients. Middendorp et al. corroborated early surgery was significantly linked to higher total motor improvement in comparison with late surgery in the overall population with SCI, while they did not differentiate patients with complete injury from those with incomplete injury. As a previous study reported that the severity of the injury is an important determinant of a patient's neurological recovery and prognosis, the spontaneous recovery of motor function in patients with AIS A grade (complete injury) is fairly limited, while AIS B patients show greater spontaneous recovery than AIS A patients [33–35]. Fawcett et al. [33] reported that over 80% of AIS A patients still maintain AIS A status until 12 months after SCI, with AIS B patients having a spontaneous recovery rate of less than 40%, while AIS C and AIS D patients have more than 60% and 95% improvement, respectively. Therefore, we assumed that AIS A (complete injury) patients have a higher risk of NeP compared to AIS B, C, and D patients, even after early surgery, but this requires further research. In addition, our present study has not reported long-term or ultimate neuropathic pain outcomes, our goal is to study NeP that occurs between 12 months after injury and analyze the impact of early surgical intervention on NeP occurrence. We believe that further follow-up is unlikely to change these conclusions, and it may be necessary to collect complete NeP evaluations at longer time points such as 2 or 5 years in future. Precisely, patients falling under AIS B, C, and D categories who underwent ultra-early surgery within 8 h of injury displayed markedly lower instances of NeP compared to those subjected to late surgery after 24 h (OR 0.148, 95% CI 0.029–0.751). Similarly, patients who underwent early surgery within 24 h of injury also experienced notably reduced NeP compared to those with delayed surgical intervention (OR 0.375, 95% CI 0.148, 0.950). These findings underscore the potential benefit of early surgical intervention in mitigating the risk of NeP, particularly among specific AIS subgroups. However, the exact mechanisms underlying this observed reduction in neuropathic pain following early/timely surgery necessitate further exploration. Post-traumatic SCI, the presence of tissue bleeding, edema, and adhesion triggers an elevation in pressure within the spinal canal, consequently setting in motion pathological and physiological processes involving ischemia and hypoxia. This, in turn, escalates the cascading reaction of secondary spinal cord injury [2, 3]. Employing an early/timely surgical approach alongside an appropriate decompression strategy can effectively curtail secondary injury [20, 32, 36]. Secondary injury including microglia activation and subsequent release of proinflammatory cytokines like TNF-α, IL-6, and IL-1β [37, 38], as well as ion channel accumulation and abnormal neurotransmitter expression [39, 40], all play crucial roles in the activation of NeP. By intervening early, it becomes plausible to avert the activation of pain pathways and the subsequent sensitization of neural circuits implicated in pain perception [41].

Additionally, our study has shed light on a compelling association, wherein patients experiencing Neuropathic Pain (NeP) were notably more prone to receiving high-dose methylprednisolone therapy during the acute phase. It is important to note that the employment of high-dose methylprednisolone is presently discouraged due to its significant systemic adverse effects [41]. Nevertheless, this intriguing finding calls for an in-depth investigation to ascertain whether the administration of methylprednisolone influences the development of NeP or merely reflects the outcome of more severe injuries.

This study represents the attempt at a comprehensive exploration of the factors underlying NeP in patients with traumatic SCI. The findings unveiled that delayed or late surgery may emerge as a noteworthy risk factor contributing to the presence of NeP. However, it is imperative to acknowledge the inherent limitations inherent in this study. Primarily, its retrospective nature introduces biases and constraints in data collection. To corroborate these findings, prospective studies with larger sample sizes and standardized protocols are indispensable. Secondarily, in the present study cohort, neuropathic pain was screened using the DN4 questionnaire as it was an ideal and validated tool for screening neuropathic pain [25, 42], the diagnosis of neuropathic pain should essentially be based on the descriptors used by the patients and the clinical physical examination results [42], validated tools and investigations should not replace detailed clinical evaluations, which may affect the robustness of our results. Thirdly, this study omitted the specific intraoperative intricacies, such as the surgical approach, decompression techniques, and the performance of stabilization procedures. Moreover, the impact and timing of preoperative closed reduction remain unreported. Notwithstanding the surgeries conducted by dedicated surgical teams at both centers, this aspect might still potentially attenuate the robustness of our conclusions. Additionally, a thorough confirmation of the extent of preoperative spinal cord compression and the efficacy of surgical interventions in fully alleviating compression or ensuring effective spinal stabilization were not specifically ascertained. Thirdly, the study lacked objective indicators for evaluating neuropathic pain, including sophisticated neurophysiological techniques like nerve conduction studies, the measurement of somatosensory-evoked potentials (SEPs), and trigeminal reflexes. These techniques play a pivotal role in confirming, localizing, and quantifying central and peripheral sensory conduction damage. Hence, future investigations should incorporate uniform pain assessment tools to ensure methodological consistency and bolster reliability.

## Conclusion

Our study proposes that incomplete AIS B, C, and D patients with acute traumatic SCI would benefit from surgical intervention performed within 8 and 24 h from time to injury, with a reduced risk of developing neuropathic pain compared to those who undergo surgical intervention later than 24 h. These findings provide evidence for the necessity of early surgical intervention, provide information for clinical decision-making, and strengthen the overall management of traumatic SCI. Efforts should be made to streamline the initial care of acute traumatic SCI patients to further reduce the time between injury and surgical intervention. Further research is warranted to elucidate the underlying mechanisms and potential therapeutic interventions to mitigate neuropathic pain in SCI patients.

### Transparency, rigor, and reproducibility

This study was not formally registered because this was a non-interventional study [1]. The analysis plan was not formally pre-registered, but the team member with primary responsibility for the analysis certifies that the analysis plan was pre-specified [2]. A sample size of 607 subjects was planned based on availability of subjects [3]. Participants were not told the results of their prognostic assessments [5]. Final clinical outcome assessments and adjudications were performed by team members blinded to relevant characteristics of the participants [5]. All surveys and questionnaires used to develop prognostic models are available from the authors. The key inclusion criteria (e.g., primary diagnosis or prognostic factor) are established standards in the field. Replication by the study group was performed as part of this study [11]. Both the original measures of statistical error rates and corrected measures of statistical error rates have been reported in the text. Data were acquired between 2021.01 and 2022.01 [6]. Data were analyzed using SPSS 26.0 (IBM, Armonk, NY, USA), while GraphPad Prism (Version 8) was utilized for data visualization. All equipment and software used to perform acquisition and analysis are widely available from IBM, Armonk, NY, USA [7]. De-identified data from this study are accessible from the corresponding author upon reasonable [12]. There is no analytic code associated with this study [13]. This paper will be published under a Creative Commons Open Access license, and upon publication will be freely available at https://www.liebertpub.com/loi/neu [14].

### Supplementary Information

**Additional file 1: Table S1.** Severity and neurological level of injury.

## Abbreviations

SCI: Spinal cord injury
NeP: Neuropathic pain
AIS: American Spinal Injury Association Impairment Scale
ISS: Injury Severity Score
QOL: Quality of life
DN4: Douleur Neuropathique 4 Questions
