# Supplementary material for: The impact of time from injury to surgery on the risk of neuropathic pain after traumatic spinal cord injury
Source: J Orthop Surg Res. 2023 Nov 11;18:857. doi: 10.1186/s13018-023-04355-7 (PMC10638760; doi:10.1186/s13018-023-04355-7)
Supplement: Supplementary file 1 — Additional file 1: Table S1. Severity and neurological level of injury. [file 13018_2023_4355_MOESM1_ESM.docx]

Table S1. Severity and Neurological Level of Injury.

|  |  | **Neurological level** | | | | |
| --- | --- | --- | --- | --- | --- | --- |
| **AIS grade** | **Neuropathic Pain /Total** | **High cervical**  **(C1–C4)** | | **Low cervical**  **(C5–T1)** | **Thoracic**  **(T2–T10)** | **Thoracolumbar**  **(T11–L2)** |
| A | 67/112 | 28/49 | 19/31 | | 8/14 | 12/18 |
| B | 18/49 | 3/11 | 10/25 | | 2/6 | 3/7 |
| C | 32/75 | 9/22 | 17/34 | | 3/9 | 3/10 |
| D | 38/84 | 10/19 | 22/46 | | 1/7 | 5/12 |
| 320 | 155/320 | 50/101 | 68/136 | | 14/36 | 23/47 |

AIS ASIA Impairment Scale.
